# Effect of the Neurofeedback-EEG Training During Physical Exercise on the Range of Mental Work Performance and Individual Physiological Parameters in Swimmers

**DOI:** 10.1007/s10484-020-09456-1

**Published:** 2020-03-30

**Authors:** Mirosław Mikicin, Anna Mróz, Magdalena Karczewska-Lindinger, Karolina Malinowska, Andrzej Mastalerz, Marek Kowalczyk

**Affiliations:** 1 Józef Piłsudski University of Physical Education, Marymoncka 34, 00-968 Warsaw, Poland; 2 Department of Molecular and Clinical Medicine, Institute of Medicine, University of Gothenburg, Gothenburg, Sweden; 3 Center for Health and Performance, Department of Food and Nutrition and Sport Science, University of Gothenburg, Gothenburg, Sweden

**Keywords:** Mental work curve, Body composition, Physical capacity, EEG, EMG

## Abstract

The aim of the study was to demonstrate the effects of the Neurofeedback-EEG training during physical exercise on the improvements in mental work performance and physiological parameters. The study examined seven swimmers based on the following anthropometric measurements: body height, body mass and body composition. The Kraepelin’s work curve test, EEG and EMG during physical exercise were also performed. The athletes followed 20 Neurofeedback-EEG training sessions on the swimming ergometer for 4 months. Most mean indices of partial measures of the work curve were significantly modified (p < 0.05) following the Neurofeedback-EEG training. Mean level of maximal oxygen uptake in study participants was over 55 ml/kg/min, with statistically significant differences documented between the first and the second measurements. No significant differences were found in the fatigue rate between the measurements 1 and 2. The improved mental work performance following the Neurofeedback-EEG training facilitates optimization of psychomotor activities.

## Introduction

It is generally accepted (Gramann et al. 2010; Gwin et al. 2011; Lau et al. 2012) that humans use multiple strategies for locomotion and motor control and are capable of controlling the rhythmic muscle activity. The effectiveness of the Neurofeedback method in inducing changes in behaviors is evaluated in particular through the measurement of the speed and accuracy of the planned action (Saleh et al. 2006). Similar criteria were used in evaluation of mental performance in the beginning of the twentieth century. Kraepelin (1922) attempted to find the causes of individual differences in labor effectiveness and drew a ‘work curve’ where he took into consideration a number of positive (motivation) and negative (fatigue) indices that determined the level of this effectiveness. It turned out that two indices of mental work curve (number of operations in the first three-minute time period and total number of addition operations) were connected with EEG in the beta 2 band which was inhibited during the Neurofeedback-EEG during exercise. The profile of the work curve has been also shown (Arnold 1975) to change with time (in the period of up to eight months) and provide a specific characterization of the person examined. There are a number of areas (Kerick et al. 2004; Monastra 2005; Hanslmayer et al. 2005; Gruzelier 2008; Besserve et al. 2008) where the Neurofeedback-EEG training offers the most promising opportunities, including improved concentration on the task, reduced fear, better control over emotions and improved physical coordination. In the sports like gymnastics, skiing, ice skating, hockey, snowboarding, combat sports and basketball, the Neurofeedback-EEG training is very likely to improve sports performance by improving psychophysical balance. Some studies (Yang and Gorassini 2006; Gorassini et al. 2009) have demonstrated, that high EEG and EMG activity recorded simultaneously during walking and running by means of independent analysis of the components may provide more insight into the motion control by the cerebral cortex and provide useful information about brain activity during walking and running in competitive settings.

In our study, we used the indices of the Kraepelin work curve to describe the relationships of mental performance with physical capacity and muscle work capability (EMG) in elite athletes after the Neurofeedback-EEG training during physical exercise. The aim of the experiment was to demonstrate that the Neurofeedback-EEG training during physical exercise improves mental work performance and physiological parameters in EEG and EMG.

## Material and Methods

**Fig. 1:**
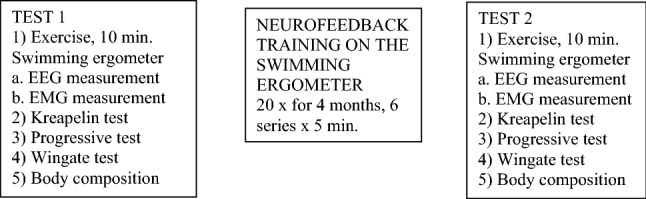
Experimental procedure

The study participants were 7 healthy swimmers aged 18 to 25 years who had not suffered lower and upper limb injuries and neurological and motor deficits before the experiment. The athletes participated in twenty Neurofeedback-EEG training sessions for 4 months. All the participants gave their written consent to participate in the experiment. The experimental design was approved by the Ministry of Science and Higher Education and were consistent with the standards defined by the Senate’s Research Bioethics Commission of the Józef Piłsudski University of Physical Education in Warsaw and standards outlined in the Declaration of Helsinki. The athletes participated in the pre-test (Test 1), followed by the Neurofeedback-EEG training sessions, with the pre-test performed after completion of all the sessions (Test 2). The procedure (experimental design) is presented in Photograph 1 and Fig. 1. The following characteristics were measured in the Test 1 and Test 2: anthropometric indices: body height and mass; body composition: fat percentage (FAT), lean body mass (LBM) and total body water (TBW). The addition test was performed to obtain the Kraepelin work curve, Holter EEG and EMG during 10 min of physical exercise. Furthermore, aerobic and anaerobic capacity of the study participants were also evaluated.

**Photo 1:**
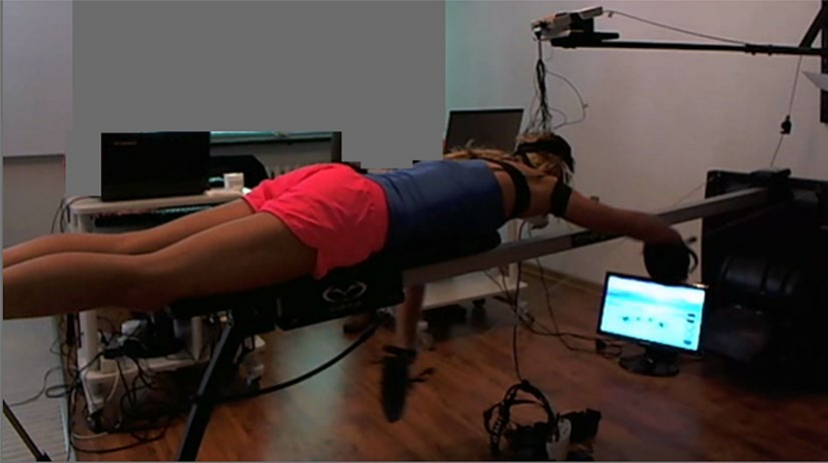
Recording (*Test 1 and Test 2*) *and* Neurofeedback-EEG trainings

### EEG During Physical Exercise, Test 1 and Test 2

The exercise EEG tests were performed in the beginning and at the end of the training sessions (before and after twenty Neurofeedback EEG sessions). Impedance of electrodes measured before recording (silver electrodes were used) was lower than 5 kOhm for each channel. After collecting the data, high-pass EEG filtering was performed for the values over 0.5 Hz. Thirty 30 EEG channels were saved for the analysis (range 2–40 Hz). Speed measurements were recorded for the participants performing crawl movements on the swimming ergometer, with lower limbs rested on the dedicated support. Electroencephalographic and electromyographic signals were recorded during 10-min submaximal exercise (each time with concentration of visual attention on the screen at the distance of around 1 m) with eyes open and closed. Brain activity recorded during electroencephalography was analyzed by means of dedicated computer software. Changes in brain activity analyzed in the experiment were related to specific activities, such as motor activity and performing a mental task. The data were analyzed with control of movement conditions.

### EMG During Physical Exercise, Test I and Test II

Surface bioelectrical activity of individual muscles was recorded by bipolar electrodes with the frequency of 1000 Hz. Raw EMG signal was filtered in the range of 20 to 500 Hz by means of an amplifier with a 16-bit analog-to-digital converter. DE-2.3 electrodes were attached along the profile of muscle bellies (deltoideus, biceps brachii, trapezius) and equipped in two silver transverse components for detection of the EMG signal on the skin surface. Sensor contacts were made of 99.9% pure silver, with length of 10 mm, diameter of 1 mm and distance of 10 mm from each other in order to achieve the optimal signal detection and cohesion levels. Alignment of these components was perpendicular to muscle fibers and allowed for maximal signal detection. The dedicated systems of signal conversion ensured a reduction of noise and transfer of a consistent EMG signal at full bandwidth of 20 to 450 Hz. The raw EMG signal was converted into a root mean square for each range of 0.0625 s for each complete section of 60 s by means of EMGWorks software (Delsys, Inc, Boston, MA). Mean value of RMS was considered to be the general measure of activity for each muscle. Mean power frequency (MPF) was evaluated through spectral analysis of the raw signal using the fast Fourier transform (FFT).

### Physical Capacity (Progressive Test, Wingate Test) and Body Composition, Test 1 and Test 2

Each exercise size was performed on different days in the morning. Aerobic capacity was evaluated based on maximal oxygen uptake and expressed in ml/kg/min. VO_2_max was measured using a direct method by means of the K4b2 Cosmed ergospirometer during progressive exercise to exhaustion. The exercise test was performed on the Monark 824 E cycle ergometer. The initial load was 1 W/kg body mass, increased every 2 min by 0.5 W/kg. The indices were recorded breath-by-breath and averaged every 5 s. Anaerobic capacity was evaluated based on power indices obtained in the 30-s Wingate test. It consisted in performing maximal exercise using a cycle ergometer (Monark 826 E). The load accounted for 7.5% body mass of the study participant. Sensors were fixed to a flywheel to record the rotation count, whereas the MCE v. 5.0 software was used to calculate parameters of power and work. The analysis was based on peak power (PP), mean power (MP), fatigue index (FI). Anthropometric indices were measured for each study participant: body height, body mass, body composition based on bioelectrical impedance (BIA) methodology by means of the body composition analyzer Tanita BC 418 MA (measurement at four leads from both feet and both hands).

### Kreapelin Test: Work Curve Test, Test 1 and Test 2

Study participants were asked to perform, within one hour, as many operations of addition of two digits displayed in adjacent columns, with the results recorded to the right. The total of proper results calculated in consecutive 3-min time periods was used to obtain the work curve. The shape of the curve, with total number of addition operations and the number of mistakes and corrections, provides the basis for interpretation of test results. The interpretation is based on Kraepelin’s studies (Arnold 1975). Measures of the work curve: (a) Performance measures: total number of addition operations (number of operations a person performed during the test, including mistakes and corrections); number of operations in the first 3-min time period (Y1, the score obtained during the first 3 min of the test, which is the indicator of previous experience in addition); maximum number of addition operations in the 3-min time period (without the first time period, which reflects the highest possible working rate of the person studied); (b) Measures of energy and persistence: percentage increase (difference between means of the first and the last four 3-min time periods expressed in percentage terms); half ratio (quotient of total number of addition operations from the period that consists of 10 last 3-min time periods (11–20) and the first ten windows (1–10), location of the maximum (3-min period number when a person studied performed the highest number of addition operations without the first period); (c) Measures of the fast adaptation and effort without self-restraint; convexity I (difference between general number of addition operations during the first four and last four time periods multiplied by mean elevation of the curve and divided by the number of time periods); convexity II (difference between the overall number of addition operations in time for the first five and last five time periods and the number of addition operations in other central ten time periods); (d) Measure of variability (or consistency), which determines indices of oscillation around the even curve (average deviation from the 3rd to 18th time period); (e) Measures of accuracy and diligence are determined by: mistake ratio (overall number of mistakes as a percentage result of general number of addition operation) and correction ratio (percentage result of overall number of addition operations); (f) Measures of additional factor are determined by the initial decline (difference between the number of addition operations in the first time period and the lowest number in the time periods 1 to 4) and duration of the decline (determined in the four first periods when the fewest addition operations were performed).

### The Neurofeedback-EEG Training During Physical Exercise Using the Swimming Ergometer

The study participants performed twenty Neurofeedback-EEG training sessions (every 7 days on the average) using a swimming ergometer, with continuation of conventional swimming training. Neurofeedback-EEG (biofeedback) training on a swimming ergometer is the process of measurement of various normal physiological parameters and real time visualization of the measurement in order to improve person's awareness of the effect of his or her actions, thoughts and emotions that on their body and develop greater control of these physiological parameters during physical exercise (Morgan and Mora 2017; Prinsloo et al. 2014). During Neurofeedback-EEG training in motion, the participants were asked to perform a task that consisted in concentration on the screen window in order to make screen change in the expected manner. Correct movement of the dolphins displayed on the screen was controlled by the feedback from an EEG amplifier when EEG signal power recorded from C3 and C4 electrodes in beta 2 band (20–30 Hz) decreased. The subjects performed this training for six times (5 min each) during a single training session. The participants relaxed after each training session by closing their eyes for 30 s. The TruScan Flex 30 Holter system was used. The EEG signal was recorded during the Neurofeedback-EEG training sessions by means of the System Flex 30 with TruScan software and the system of electrodes in the 10–20 arrangement. Resistance of electrodes was maintained at the level of below 5 kOhm and the amplifier signal was filtered within the range of from 2 to 40 Hz.

### Statistical Analysis of the Data

The means and medians were calculated for each frequency range and each area of EEG and EMG at varied movement speed. Next, the EEG and EMG spectrograms were obtained for each source and both speeds. The significance of differences in the indices of aerobic and anaerobic capacity measured during the first and the second test was evaluated by means of the Student’s *t*-test for dependent samples. Non-parametric tests were used for the statistical data analysis: the Wilcoxon signed-rank test, the Mann–Whitney *U*-test, repeated measures ANOVA, the Spearman’s R correlations and descriptive statistics.

## Results

### Anthropometric Indices, Body Composition And Physical Capacity

The values of basic anthropometric indices of the athletes analyzed in the study are shown in Table 1.

**Table 1:** Mean anthropometric indices of the study participants

Index	Age (l)	Body Height (cm)	Body mass (cm)		BMI	
			1 bad	2 bad	1 bad	2 bad
Group						
Swimmers (n = 7)	20.6 ± 1.40	182 ± 3.16	75.4 ± 4.13	74.2 ± 5.35	22.8 ± 1.10	22.5 ± 1.44

Body composition evaluated in the pre-test (before starting the Neurofeedback-EEG training sessions and after completion of the experiment) did not show statistically significant differences. Aerobic capacity was evaluated based on the VO_2_max index in the progressive test. The mean value of maximal oxygen uptake in athletes was over 55 ml/kg/min and the difference between the first and the second measurement was not statistically significant (0.6 ml/kg/min.) Power parameters measured in the Wingate test were used for evaluation of the anaerobic capacity. No statistically significant differences were observed between the first and the second measurement (Table 2).

**Table 2:** Mean anthropometric indices, body components and indices of anaerobic and aerobic capacity in athletes recorded in Test 1 and Test 2

Parameters		Test I	Test II	p
FAT	%	10.5 ± 4.89	11.2 ± 6.29	0.8335
	kg	7.8 ± 3.38	8.1 ± 4.29	0.8885
LBM	%	89.5 ± 4.89	88.8 ± 6.29	0.8335
	kg	67.6 ± 6.41	66.1 ± 7.83	0.7044
TBW	%	65.2 ± 3.86	64.9 ± 4.53	0.9148
	kg	49.5 ± 4.71	48.3 ± 5.69	0.6836
VO_2_ max	l/min	4.27 ± 0.74	4.13 ± 1.07	0.7890
	ml/kg/min	56.4 ± 8.94	55.8 ± 13.86	0.9286
P mean	W/kg	8.20 ± 0.77	8.34 ± 0.73	0.7491
P peak	W/kg	10.33 ± 1.37	10.54 ± 1.13	0.7774
Fatigue index	%	19.49 ± 5.98	20.73 ± 4.24	0.6878

### The Parameters of Mental Work Curve (Kreapelin Test) After the Neurofeedback-EEG Training

The most of mean indices that describe partial measures of the work curve were changed significantly (at the level of p < 0.05) following Neurofeedback-EEG training (Table 3). Of the three measures of mental work, we observed tendencies for improvements in the working rates in the experimental group (n = 7). Furthermore, significant changes (p < 0.05) were also found in the case of two of three parameters calculated based on the test results, energy and persistence: the difference in mean working curve elevation and first ten periods. These changes in partial measures suggest that the work curve was increasing, which might have resulted from fatigue due to the high working rate in the first half hour of the test. Both parameters of the measures of adaptation and exercise without self-restraint (convexity I and II) were reduced significantly (p < 0.05). The index of oscillation around the work curve (p < 0.05) was also reduced; it is presented as a measure of variability that points to consistency and a reduction in the level of emotional distress during the test. The increase in the correction ratio (p < 0.05), calculated as a percentage of the total number of addition operations, is interpreted as an improvement in the measure of accuracy and diligence during work. Another significant observation is the increase in the percentage of corrections that occurs with a reduction in the percentage of mistakes. Of additional measures, shortening of the duration of the initial decline (p < 0.05) was observed, which has been explained in the literature as an increase in experience and better adaptation to new situations (Kraepelin 1922; Arnold 1975).

**Table 3:** Indices of the work curve before and after the Neurofeedback-EEG training in the experimental group (n = 7)

Indices	Rank sum	Rank sum	U	Z	p
	Before nfb	After nfb			
Total number	277.00	389.00	106.00	− 1.76	0.079
First three minute	271.00	395.00	100.00	− 1.95	0.052
Period I	265.50	364.50	112.50	− 1.32	0.187
% increment	271.00	395.00	100.00	1.95	0.052*
II/I ratio	265.00	401.00	94.00	2.14	0.033*
Peak location	358.00	272.00	101.00	1.70	0.089
Convexity I	385.00	241.00	145.50	0.51	0.046*
Convexity II	372.50	234.50	122.50	1.23	0.055*
Oscillation I	351.00	256.50	144.00	0.55	0.058*
Mistake ratio	294.00	234.00	81.00	1.74	0.082
Correction ratio	263.50	402.50	92.50	− 2.18	0.029*
Initial decline	350.50	315.50	144.50	0.54	0.591
Duration of the decline	398.00	268.00	97.00	2.04	0.041*

The comparison based on the Wilcoxon signed-rank test

*Coefficient of significance p < 0.05

**Fig. 2:**
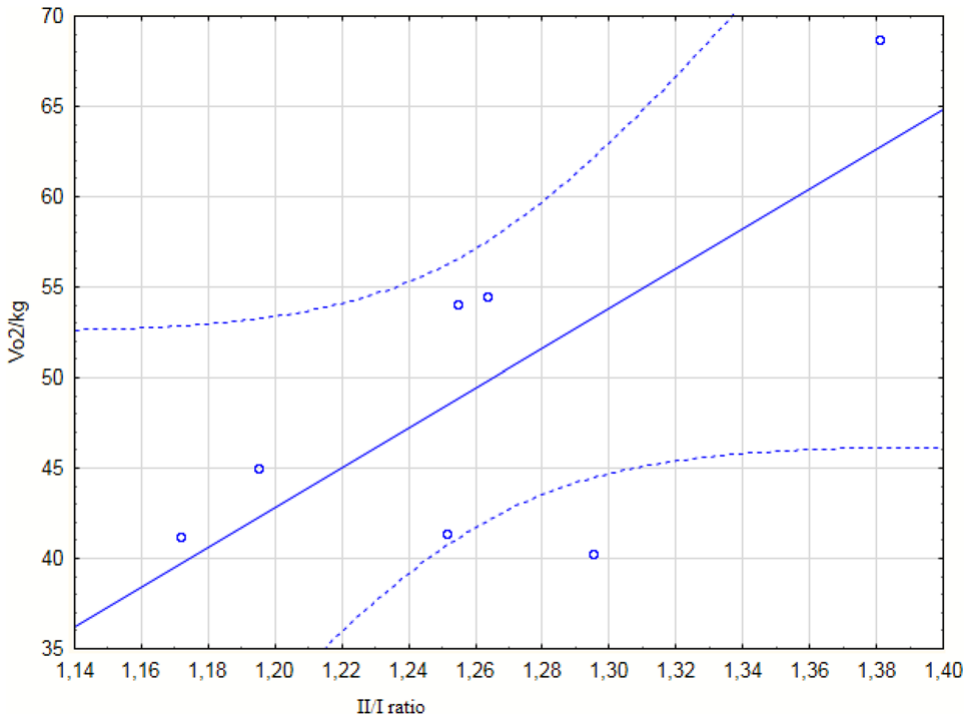
Spearman’s correlation: Ratio II:I (half ratio) with VO_2_max (ml/kg/min), r = .776, correlation coefficient p < 0.050

**Fig. 3:**
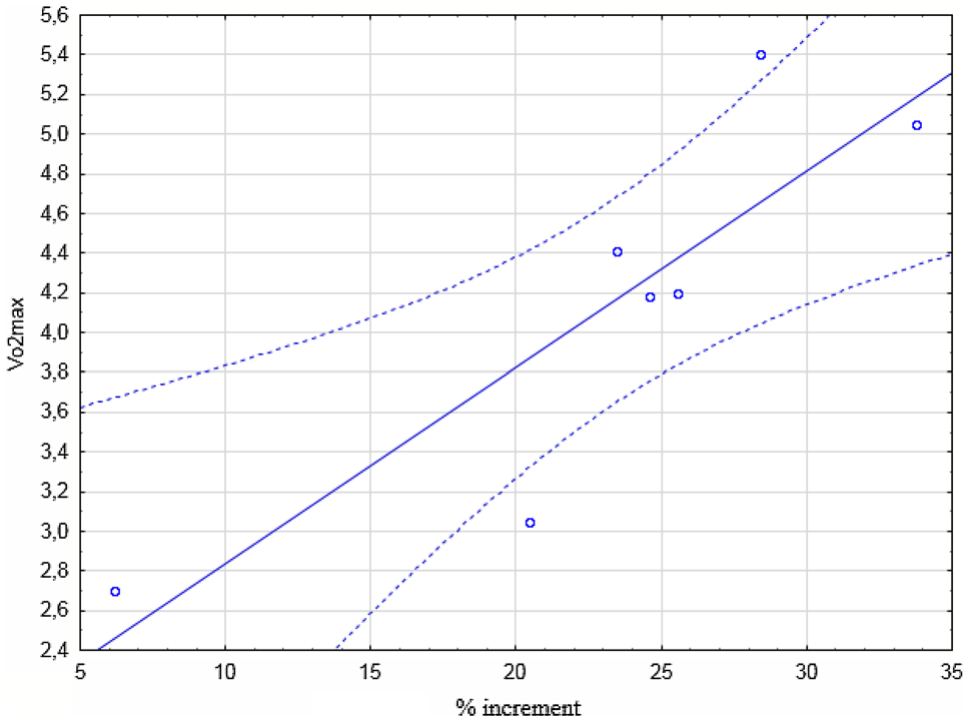
Spearman’s correlation between the percentage increase (see “Material and Methods”) and VO_2_max (l/min), r = .872, correlation coefficient p < 0.050

Measures of energy and persistence in terms of the half ratio i.e. the quotient of total number of addition operations from the period that consisted of ten 3-min time periods (11–20) and the first ten windows (1–10), which is positively correlated with the aerobic capacity index VO_2_max (ml/kg/min) (Fig. 2) and in terms of the increase i.e. the difference between the means from four first and four last 3-min periods expressed in percentage terms, which is additionally correlated with VO_2_max (Fig. 3).

### EEG, EMG During Submaximal Exercise After the Neurofeedback-EEG Training During Physical Exercise

Muscle fatigue was assessed using the slope of regression line. It characterizes the rate of changes in mean power of EMG signal spectrum (MPF) during the test exercise. The greater the magnitude of the slope, the steeper the line and the greater the rate of change. The data obtained in the study lead to the conclusion that the test exercise caused fatigue in all muscle groups analyzed in the study (see Table 4). The rate of this fatigue was similar. No significant differences were found in the fatigue rate between the measurements 1 and 2.

**Table 4:** Average value (± SD) of the slope of regression line [Hz/s] in measurements 1 and 2

Measurement	Deltoideus		Biceps brachii		Trapezius	
	Mean (Hz/s)	SD (Hz/s)	Mean (Hz/s)	SD (Hz/s)	Mean (Hz/s)	SD (Hz/s)
1	− 0.53035	1.846668	− 0.13212	1.384861	− 0.92417	1.476109
2	− 0.8999	1.808735	− 0.68396	2.023446	− 0.3807	1.5256

## Discussion

The combination of mental effort (concentration on the computer screen) and physical exercise (crawl movements performed on the swimming ergometer) led to changes in brain activity. A substantial increase in the amplitude was observed in all brain regions, especially in the initial phase of the experiment. The previous studies have found (Gwin et al. 2011) increased activity in the sensorimotor cortex during submaximal exercise, and, in several registers, also in the dorsolateral prefrontal cortex and premotor cortex. It is likely that greater increase in the EEG power of the sensorimotor cortex would occur during recruitment of stronger muscles of the limbs (e.g. in strength sports). Therefore, the analysis of the swimming movements may be used in the future to identify correlations between muscle recruitment and dynamics of brain activity during swimming.

Analysis of the distribution of bioelectrical activity of the cerebral cortex connected with the rate of movements and events connected with desynchronization (ERD) and synchronization (ERS) of cortical EEG rhythms. The activity of posterior parietal and occipital cortices is likely to be linked with visual-motor integration and coordination. Changes in voltage were insignificant (i.e., the periods of substantial spectral fluctuations occurred for different speeds). One exception was that the high-fluctuation gamma band was present in the right sensory cortex in two phases of the exercise test (in the left sensorimotor cortex, frontal cortex, and posterior parietal cortex). This is consistent with the convincing findings which showed that bilateral coordination does not recruit the left hemisphere but it controls the position of the limb and body posture (Serrien et al. 2006). Fluctuation of the voltage in the right and posterior parietal cortex may be consistent with visual-motor integration responsible for concentration during movement—during the movement and during performance of the Kreapelin test, where a significant improvement was observed.

## Conclusions

The improvements in maximum performance following the Neurofeedback-EEG training had an effect on optimization of psychomotor activities. These changes were not substantial but they are likely to point to the tendencies for attention engagement during motor activities. Therefore, the level of amplitudes of beta1 bands following the Neurofeedback-EEG training during physical exercise may suggest the tendencies for maintaining energy and consistency in action.
